# Electrochemical Biosensors as a Novel Platform in the Identification of Listeriosis Infection

**DOI:** 10.3390/bios13020216

**Published:** 2023-02-01

**Authors:** Leila Mehrannia, Balal Khalilzadeh, Reza Rahbarghazi, Morteza Milani, Gulsah Saydan Kanberoglu, Hadi Yousefi, Nevin Erk

**Affiliations:** 1 Department of Medical Biotechnology, Faculty of Advanced Medical Sciences, Tabriz University of Medical Sciences, Tabriz 51666-14733, Iran; 2 Stem Cell Research Center, Tabriz University of Medical Sciences, Tabriz 51666-14733, Iran; 3 Department of Applied Cell Sciences, Faculty of Advanced Medical Sciences, Tabriz University of Medical Sciences, Tabriz 51666-14733, Iran; 4 Department of Chemistry, Faculty of Science, Van Yuzuncu Yil University, Van 65080, Turkey; 5 Department of Basic Medical Sciences, Khoy University of Medical Sciences, Khoy 58167-53464, Iran; 6 Department of Analytical Chemistry, Faculty of Pharmacy, Ankara University, Ankara 06560, Turkey

**Keywords:** *Listeria monocytogenes*, electrochemical biosensors, bacteriosensors, infection, nanomaterial, listeriosis

## Abstract

*Listeria monocytogenes* (L.M.) is a gram-positive bacillus with wide distribution in the environment. This bacterium contaminates water sources and food products and can be transmitted to the human population. The infection caused by L.M. is called listeriosis and is common in pregnant women, immune-deficient patients, and older adults. Based on the released statistics, listeriosis has a high rate of hospitalization and mortality; thus, rapid and timely detection of food contamination and listeriosis cases is necessary. During the last few decades, biosensors have been used for the detection and monitoring of varied bacteria species. These devices are detection platforms with great sensitivity and low detection limits. Among different types of biosensors, electrochemical biosensors have a high capability to circumvent several drawbacks associated with the application of conventional laboratory techniques. In this review article, different electrochemical biosensor types used for the detection of listeriosis were discussed in terms of actuators, bioreceptors, specific working electrodes, and signal amplification. We hope that this review will facilitate researchers to access a complete and comprehensive template for pathogen detection based on the different formats of electrochemical biosensors.

## 1. Introduction

Bacteria are responsible for 40% of the annual mortality rate [1,2]. It has been indicated that both bacteria and their toxins can contaminate food sources and water [3]. It has been estimated that nearly 48 million people are diagnosed with different forms of foodborne diseases each year, leading to 128,000 hospitalizations and a mortality rate of 3000 cases in the United States [4]. Along with these comments, rapid and precise identification of the causal agent plays a crucial role in the prevention of bacterial infections [5]. Since the number of bacterial particles in contaminated foods is limited, successful identification and detection is more challenging in bio-fluids, foods, and other aqueous substances [6,7].

*Listeria monocytogenes* (L.M.) is a gram-positive bacterium with a wide distribution in water, soil, and crude or prepared foods [8,9]. L.M. particles can be transmitted by ready-to-eat foods due to their high shelf life and direct consumption [7,10]. Microbiological analyses have revealed that L.M. can grow easily in a wide range of temperatures (3 to 45 °C) and pH values (5.4–9.6) with high levels of salt [11]. Listeriosis is caused by L.M. after the ingestion of contaminated foods and byproducts. Infection with L.M. contributes to the promotion of several pathological conditions and the involvement of different organ types, resulting in a mortality rate of 20–30%. Common clinical findings in patients with listeriosis are septicemia, meningoencephalitis, preterm newborns, and abortion [7,12,13]. Although the incidence of listeriosis is high in immunocompromised cases, older adults, and pregnant females, this bacterial agent can affect individuals with normal immune system function [7,9].

Culturing it in microbiology media is thought of as a commonly available method for the expansion and detection of L.M. Culturing is precise and helps with the differential diagnosis, but the procedure is very laborious and time-consuming (up to 5–7 days) [6,8,14,15]. Polymerase chain reaction (PCR) and enzyme-linked immunosorbent assay are other available approaches used for the diagnosis of L.M. Despite the relatively appropriate sensitivity and specificity, these methods are expensive, and their use depends on experienced technicians and sample preparation [9,16]. Therefore, a rapid and reliable strategy should be developed for monitoring listeriosis. In recent decades, we have witnessed the emergence of numerous strategies for rapid L.M. identification using specific detection methods [7]. Biosensors are a powerful diagnostic strategy for the detection of various analytes [17,18]. Some challenges in the development of electrochemical biosensors include stability; the balance between sensitivity and response time; loss of true activity in real samples; and integration turbulence, which are problematic in the application of electrochemical sensors from the lab to the clinical setting [19]. Finding solutions to these problems could turn electrochemical biosensors into in situ detection devices. This review aimed to highlight the potency of electrochemical and biosensors in the detection of L.M. Herein, the eligibility of electrochemical biosensors is highlighted in terms of L.M. particles, genomics, and proteomics in different environments.

## 2. Electrochemical Biosensors and L.M. Diagnosis

Biosensors are analytical devices with the potential to sense biological [20,21,22,23,24,25] and chemical [26,27] analytes via the production of signals [7]. These sophisticated platforms are made of several parts, as follows: sensing elements, converters, signal processors, and detectors [7]. Notably, biosensors can expedite the analytical process using small sample sizes, making them powerful and highly sensitive devices [28]. To date, various types of biosensors have been developed according to the modes of analytes such as thermometric biosensors, electrochemical biosensors, optical biosensors, and gravimetric biosensors [9]. One of the challenges in thermometric biosensors is assay specificity. This feature causes several difficulties in the differentiation of specific responses from nonspecific responses [29]. Optical biosensors, such as fluorescence-based biosensors, rely on the presence of expensive fluorescent-sensitive equipment [30].

Compared to different biosensor types, electrochemical biosensors with greater sensitivity are more efficient, time-consuming, and user-friendly, and have portable features [31,32,33]. These types of biosensors can be used for the in situ detection of microorganisms in muddy samples due to a lack of preparation steps and a need for external reagents [1,9,28,34,35]. The process of detection is based on measuring changes in current or voltage in the presence of a specific analyte [35]. Recent progress in the field has led to the advent of different electrochemical techniques for evaluating L.M., such as amperometric, potentiometric, voltammetric, and impedimetric methods [35].

### 2.1. Amperometric Biosensor

**Figure 1:**
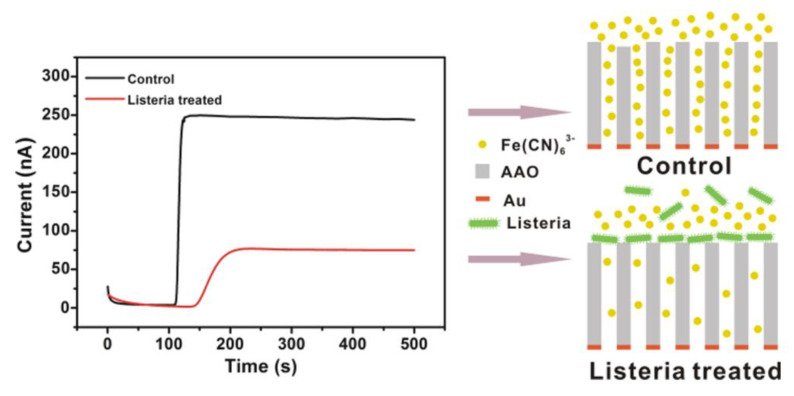
A schematic illustration of an electrochemical amperometric biosensor developed for *Listeria* detection. Copyright (2022) American Chemical [36].

In the amperometric actuator model, the current is produced through an oxidation–reduction reaction with the simultaneous transfer of electrons between chemical species [9]. Previously, Zhou and co-workers designed an amperometric method for analyzing L.M. particles (Figure 1). They applied permeable anodic (aluminum oxide) film conjugated with a specific aptamer for the evaluation of bacterial particles. They reported a significant reduction in current based on the penetration of the probe from the film that was blocked with L.M. bacilli onto the detection layer. This study exhibited a sensitivity detection limit of 10^2^ CFU/mL during a 10-min period [36].

### 2.2. Potentiometric Biosensors

**Figure 2:**
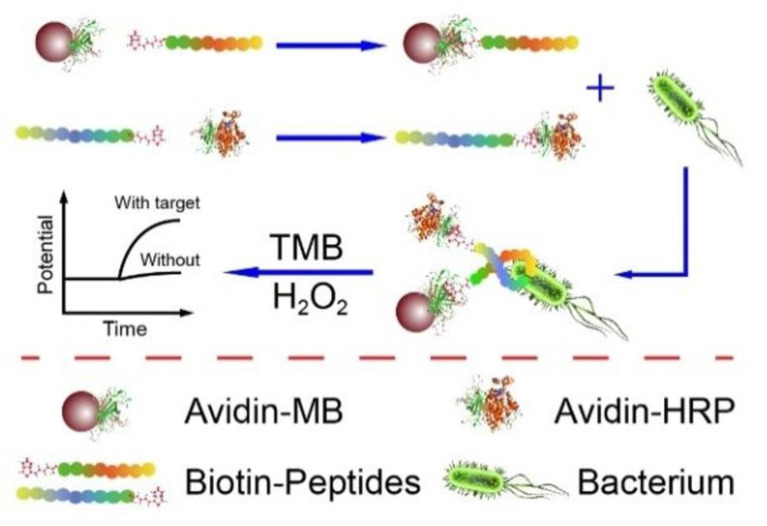
A potentiometric electrochemical biosensor composed of the biotinylated anti-bacterial peptide as a recognition element, magnetic beads as a concentrator of the bacterial cell, and enzymatic detection indicator. Copyright (2022) ACS publications [38].

In this type of biosensor, the current is measured according to the potential created in the working electrode [37]. Enguang Lv et al. developed a potentiometric technique for L.M. detection. In this study, a biotinylated recognition peptide was conjugated with magnetic connectors and an enzymatic detection indicator for the purpose of sensing L.M. in the sandwich route (Figure 2). In the enzymatic detector, a detection probe similar to horseradish peroxidase (HRP) was used. The developed system had the potential to sense bacilli with a limit of detection of 10 CFU/mL and 1.0 × 10^2^ to 1.0 × 10^6^ CFU/mL in a linear range [38].

### 2.3. Impedimetric Biosensors

This technique records changes in conductivity and resistance between the working electrode and the electrolyte [9]. In an experiment conducted by Kashish and co-workers, they detected L.M. particles via a genosensor based on an impedimetric technique. To this end, N, N′-(3-dimethyl aminopropyl-ethylcarbodiimidehydrochloride) (EDC), and N (hydroxysuccinimide) (NHS) were used to immobilize single-strand DNA bioreceptors on a conductive polymer (poly(5-carboxy indole)) [39]. When complementary target DNA existed in the samples, a hybridization reaction was promoted, leading to changes in the impedance. These authors claimed that the developed system was able to measure bacterial particles using a label-free approach with a linearity range of 1 × 10^−4^ to 1 × 10^−12^ M [39].

## 3. Type of Bioreceptor

Antibodies, aptamers, DNAs/RNAs, and enzymes were common materials used for bioreceptor applications [34,40].

### 3.1. Antibody-Based Bioreceptor (Immunosensors)

It is believed that immunological biosensors can sense the targeting of analytes using different strategies. For example, some of the immunological biosensor types are sensitive to mass changes, whereas other types can sense the changes in optical properties or electrochemical changes in applied transducers [7]. In this scenario, the electrochemical immunosensors receive considerable attention due to their exceptional sensitivity and fast detection rate [7,41]. The common technique used for the electrochemical immunosensors platform is the sandwich method. In this approach, primary antibodies are fixed on the electrode surface, and secondary antibodies are attached to specific reporters with the ability to calculate the number of analytes [41]. Previous experiments have used both polyclonal and monoclonal antibodies for the detection of L.M. Notably, polyclonal antibodies are reasonably priced, with higher sensitivity and less specificity compared to monoclonal antibodies [9]. Chen et al. prepared an immunosensing device that incorporates a monoclonal anti-*Listeria*-magnetic isolation process, as well as urease activity, using an impedance technique. They detected L.M. in different samples containing different ranges of bacterial colonies (3.0 × 10^1^ to 3.0 × 10^4^ CFU/mL). Based on the data, the lower limit of the detection system was 300 CFU/mL. Considering the application of bare electrodes, the sensor represented impressive recyclability [42].

### 3.2. Cytosensor

Agni et al. designed a cell-based immunosensor (cytosensor) for the detection of L.M. using engineered monkey cells harboring anti-LM p60 antibodies on their outer membrane surfaces [43]. The potentiometry technique revealed a minimum detection range of 2 log CFU/mL, with an accuracy of 88%. This system can differentiate the presence of L.M. from that of other pathogenic bacteria such as *Escherichia coli* and other *Listeria* species [43]. Despite the existence of limitations to the application of T cell-based biosensors compared to molecular sensors, the applied system was able to measure target analytes in an environment similar to in vivo conditions [44]. In a study conducted by Silva et al., an electrochemical immunosensor was applied to monitor L.M. particles in food products using spiked milk [45]. For this purpose, an anti-p60 antibody was stabilized on carbon screen-printed electrodes, and an alkaline phosphatase-conjugated secondary antibody (a signal amplifier) was used in a sandwich pattern. Data indicated a detection limit of 1.5 ng/mL for the target protein in a detection period of <3 h [45]. Since environmental parameters such as pH and temperature can change the affinity of antibodies and antigens, the bulk application of bioreceptors in the laboratory setting and further translation into the clinical setting is challenging [1]. Some antibody-based biosensors for detection of *Listeria* are demonstrated in Table 1.

### 3.3. Enzymatic Biosensors

Enzymes are biocatalysts that facilitate the conversion of specific substrates to products [28]. An oxide/reduction reaction causes electrochemical changes that can be detected by biosensors [28]. Because of their intrinsic high catalytic function and precise ligand interaction, enzymes can be used in biosensors. Despite these advantages, enzymes are rather unstable and influence the structure of biosensors [1]. In addition, enzymes possess limited reusability with simultaneous strong alternative interaction properties that necessitate the pre-concentration of L.M. [7]. Tolba and colleagues developed an impedance-based electrochemical biosensor using L.M-specific bacteriophage peptidoglycan hydrolases (endolysins)-modified screen-printed gold electrodes to pre-concentrate and recognize bacterial particles. This enzyme has an exhibited high-rate specific binding capacity to target bacteria and promote their lysis. The data indicated an enrichment efficiency of 86–99%. Bacterial lysis caused impedance changes. The developed biosensor showed a detection limit of 1.1 × 10^4^ and 10^5^ CFU/mL in the culture medium and milk sample, respectively [58].

### 3.4. Nucleic Acid-Based Bioreceptor

Deoxyribonucleic acid (DNA) is commonly used in the development and design of several electrochemical biosensor types using different synthesis approaches. To this end, DNA is synthesized in a laboratory with a known sequence, which is called an aptamer [59]. By comparing data from different experiments with varied limits on detection rates, methods relying on nucleic acid can be found to be more precise compared to approaches that target immunological reactions [9].

#### 3.4.1. Aptasensors

**Figure 3:**
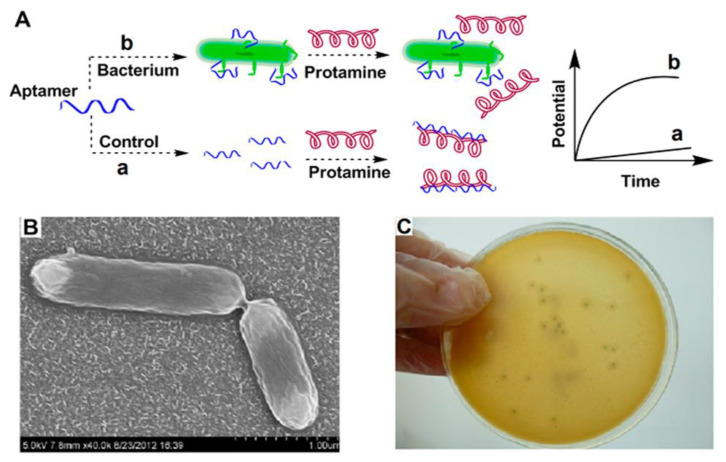
An example of an aptasensor binding with a *Listeria* surface protein (Internalin A). Protamine is a positive-charge substance that reacts with aptamer alone and with a bacteria–aptamer complex. Each of these reactions causes specific potential changes. The polymeric polycation-sensitive membrane can detect the presence of this composition. (**A**) Mechanism of biosensor, (**B**) Scanning electron microscope (SEM) illustration of bacteria and (**C**) Image of the cultured bacteria. Copyright (2022) from ACS publications [11].

Antibodies are important components of immunological experiments for the fabrication of biosensors. Unfortunately, antibodies possess short half-lives and their function is affected by environmental parameters such as temperatures and pH values, leading to denaturation and misfolding [7]. Along with these comments, the production, purification, and preservation of antibodies face some challenges. For example, it is difficult to produce stable and similar anti-serums due to the need for animal immunization and the development of hybridoma cell lines. The process of immunization in animals is long, and maintenance of hybridoma cell lines is difficult [6,7]. Aptamers or “chemical antibodies” are a special class of single-strand oligonucleotides or synthetic peptides that are capable of specifically interacting with a target antigen due to their exceptional three-dimensional structures [9,34]. The structural properties of aptamers and chemical construction methods have made aptamers a great alternative for circumventing antibody limitations [60,61]. In comparison with antibodies, aptamers have several benefits, as follows. The modification of aptamers is easy, and their synthesis is achieved using artificial approaches [7,62]. The selection of an interest aptamer was carried out via an in vitro method by using the systematic evolution of ligands with an exponential enrichment method, which takes less time than the favored method of antibody selection (several weeks vs. a few months) [7,63]. Furthermore, mass production of aptamers is affordable and these elements can bind to a variety of substrates, from biomolecules to cells, while antibodies target immunology-associated components [9]. Due to the structural stability and bioactivity of aptamers after being exposed to thermal shock, aptamers have superiority to antibodies in the fabrication of biosensors. In contrast to antibodies, the structural changes of aptamers are reversible. The duplicability of the aptamer is excellent. They have long half-lives at room temperature without any change in their function; therefore, diagnosis using aptasensors is cost-effective [7,9,34,59]. Regarding these features, researchers have used aptamers as biomarkers in many studies. Ding et al. designed a potentiometric biosensor using an aptamer against internalin A to detect virulent L.M. (Figure 3). In this work, protamine was used to interact with a free aptamer that had not been bound to internalin A. A protamine-aptamer complex was detected with an electrode of the polycation-responsive membrane. Using this biosensor, L.M. could be sensed down to 10 CFU/mL [11]. Daniela A. Oliveira and colleagues designed an aptasensor to detect *Listeria*. They used a co-deposition technique to modify the electrode surface with nanoplatinum and alginate thiomers. This structure (ALG-thiomer/Pt nanobrush) formed a pH-sensitive and electroactive surface. In this work, aptamer acts as a bioreceptor to capture internalin A protein. The calculated detection limit reached 5 CFU mL^−1^ and the assay time was about 17 min. The biosensor showed great selectivity with other gram-positive bacteria [64]. Chen et al. used a 3D graphene composite doped with zinc nitrogen (ZnO-3DNGH) to modify the platinum electrode. They immobilized the amino-terminated aptamer on the modified electrode. Their designed biosensor utilized the electrochemical luminescence method to detect *Listeria* in pork and milk samples. The detection limit in this method was 5 CFU/mL [65].

#### 3.4.2. Genosensors

**Figure 4:**
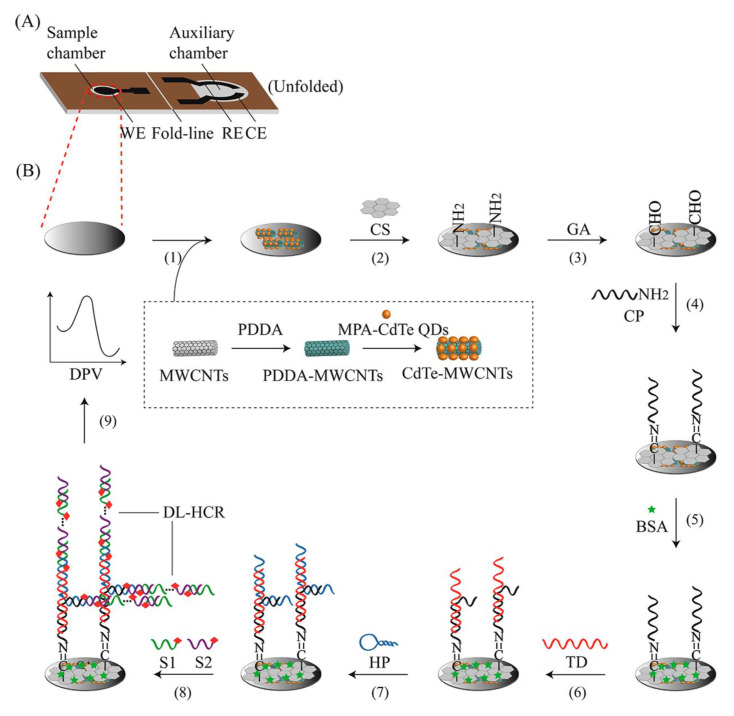
A schematic illustration of a genosensor with integrated electrodes that sense target DNA using a double linear hybridization chain reaction (DL-HCR) technique and short signal DNA probe pairs. (**A**) Graphical presentation of the developed chip and (**B**) Preparation steps of the genosensor. Copyright (2022) from ACS publications [70].

Biosensors with natural DNA molecules as recognition elements are called genosensors [59]. Genosensors provide a rapid method for the recognition of various analytes, such as pathogens [66]. This technique relies on the reaction of DNA with complementary nucleic acid sequences. In these biosensors, a probe of single-stranded DNA acts as a recognition element that forms a double strand with target DNA with exceptional performance and specificity. Genosensors can be used as probes for the determination of virulence factor genes, such as the hemolysin A (hlyA) gene, in L.M. Upon attachment of stabilized DNA to the target sequence, a converter converts the reaction to the measurable signal [66,67]. In electrochemical genosensors, identification of the hybridization reaction is conducted either by a direct method, by utilizing intercalating compounds that bond to double-strand DNA, or by using a reporter probe [68]. Kashish et al. applied impedance spectroscopy with a glassy carbon electrode modified with platinum nanoparticles. They used 24-mer single-strand DNA to recognize the L.M. hlyA gene in milk samples, with a sensing range of 1 × 10^−12^ M to 1 × 10^−4^ M [69]. In another experiment, Jiang and co-workers designed a potentiometric genosensor for L.M. detection in milk samples (Figure 4). They performed an assay using CdTe QDs/MWCNTs nanostructure-modified carbon ink in a solid wax matrix screen-printing electrode [70]. To amplify electrical signals, a double linear hybridization chain reaction (DL-HCR) was used. Authors used 8.74 fM with the synthetic target DNA, 0.039 ng/μL with the digested *Listeria* genome, 1.64 × 10^4^ CFU/mL in the milk samples, and 11 CFU/mL in the milk samples followed by PCR as detection limits [70]. Ebana et al. designed an amperometry-based electrochemical genosensor, with silica and magnetic particles as the platform. They immobilized biotinylated-long DNA strands (PCR product) of the prfA (transcriptional activator of the virulence factor) gene on a platform as a capture. They used streptavidin–horseradish peroxidase to detect the presence of a PCR amplicon instead of electrophoresis [71]. Recently published genosensors for the evaluation of *Listeria* are presented in Table 2.

#### 3.4.3. CRISPR/Cas-Based Electrochemical Biosensor

The clustered, regularly interspaced, short, and palindromic repeats/CRISPR-associated nuclease 9 (CRISPR/Cas 9) is a type of adaptive immune system that is seen in bacteria and archaea. It is a fragment of the genome of an invasive organism (bacteriophage, etc.) that integrates with microbial genomes. Transcripts of these inserted fragments (RNA-guided) can recognize inactive related targets by endonuclease activity [82,83]. Fan Li et al. developed an electrochemical biosensor that used the CRISPR/Cas12a system. The designed platform used the cleavage ability of guide CRISPR RNA (crRNA) to recognize single-strand DNA and cut methylene blue-tagged ssDNA. They reported 0.68 aM of extracted genomic DNA and 26 CFU/mL of L.M in culture media as the limits of detection [30].

### 3.5. Bacteriophages as Bioreceptors

Bacteriophages are a group of viruses that attack and kill bacteria [84,85]. They can also distinguish between live bacteria and dead cells [86]. Zolti and co-workers developed a bacteriophage-based biosensor. They modified carbon nanotubes with quaternized polyethyleneimine and used it as a matrix to immobilize the P100 bacteriophage. Their platform showed 8.4 CFU/mL as the detection limit [86].

## 4. Category of Working Electrodes

### 4.1. Interdigitated Electrode 

Due to the high capacity of the interdigitated electrodes, biosensors based on these electrode types showed great sensitivity [87]. Sidhu et al. developed an impedance biosensor for the detection of *Listeria monocytogenes*. They used platinum interdigitated microelectrodes (Pt-IDEs) decorated with L.M. aptamers (thiolated aptamer of internalin A gene) as working electrodes. The results were obtained using the EIS method at a range of 10 to 10^6^ CFU/mL. This biosensor exhibited a detection limit of 5.39 ± 0.21 CFU/mL for 17 min [88].

### 4.2. Screen-Printed Electrodes

These electrodes are promising platforms with a great capacity for use in electrical biosensors as transducers. Their main advantages include availability, portability, and low-volume sample loading, making them appropriate for on-site detection [89]. Bifulco and co-workers used screen-printed gold electrodes that copped with the mercaptan-activated self-assembled monolayer (SAM) to detect the internalin A gene. Their designed platforms showed significant spasticity for L.M. versus other *Listeria* species (*p* = 0.0016), and the concentration of applied probe and DNA was 100 ppm [72].

### 4.3. Glassy Carbon Electrode (GCE)

GCE is one of the most utilized working electrodes. This electrode is functionalized with a variety of materials, including chitosan-dispersed platinum nanoparticles [69].

### 4.4. Gold Electrode (AuE)

AuE can be applied as electrodes in biosensors at bulk films and thin films by a variety of methods. Because of their great electron kinetics and stability, as well as their simple capability to functionalize, AuEs have received more attention in the era of biosensors [90]. It has been suggested that gold can preserve its natural solid structure during a broad range of potential changes with varied pH indices. Various techniques can be applied to modify the electrode surface of gold [90]. Wu et al. performed impedimetric assays for the detection of L.M. using mercaptan, NHS, and EDC-activated AuE coated by the listeriolysin O gene [73]. Routinely applied working electrodes for the quantification of *Listeria* are summarized in Table 3.

## 5. Method of Signal Amplification

One way to increase the sensitivity of biosensors is to amplify the signal resulting from the reaction between the target material and the bioreceptor [92]. Different methods were applied to functionalize the surfaces, such as lasers [93].

### 5.1. Enhancement of Sensitivity Using Labeling or Capturing Methods 

#### 5.1.1. Enzymes as a Label

Electrochemical-based signal amplification strategies rely on enzymes and oxidoreduction methods [94]. Hajdukiewicz et al. utilized glucose oxidase to amplify the detection signal in biosensors with a graphite electrode. This amperometry technique was applied to recognize the biotinylated L.M. ssrA gene with a 20-mer aminated ssDNA. The detection process was based on the interaction of the DNA probe with a biotinylated target sequence. This reaction was reported by glucose oxidase–avidin D in the presence of glucose in a ferrocene methanol-based medium with a detection limit of 0.2 nM [95]. Recently, researchers have paid expansive attention to nanozymes, as well as organic nanoparticles with metal nanoparticles, to amplify detection signals [96,97,98,99]. In comparison with other metal nanomaterials, Au- and Pt-based nanomaterials possess great stability and exceptional catalytic potency [100]. Interestingly, the Au–Pt(core-shell)-based nanozyme (the nanostructure consisting of a composition of Au and Pt that catalyze a reaction) has been introduced as a capable alternative bioenzyme for signal amplification [100].

#### 5.1.2. Electroactive Agents as a Label

The electrochemically labeled probes (DNA probes) have been widely applied in detection approaches. These materials are inorganic or organic forms, and they present a strong redox response associated with a hybridization reaction. Inorganic forms, such as metals (for example, gold nanoparticles, as discussed below), and organic forms, such as methylene blue and ferrocene (Fc), have been commonly utilized [101]. A summary of electroactive labels for the detection of *Listeria* is presented in Table 4.

#### 5.1.3. Nanomaterials as a Label

Bare electrodes suffer from some drawbacks, including, for example, loss of sufficient electrical activity and unpredictable surface structures [102]. The modification of bare electrodes plays a fundamental role in the reproducibility, sensitivity, and specificity of biosensors [103]. Thus, a variety of functionalized compounds, such as nanomaterials, was utilized to design efficient electrochemical biosensors [102,104]. For this aim, carbon-based nanomaterials, such as graphene and metal nanostructures, were applied [105].

A combination of nanomaterials and electrochemical biosensors has been used for signal amplification strategies. Nanoparticles provide an excellent opportunity for developing point-of-care and scale-down tools for medical applications [28]. Nanomaterials are extracted from natural sources or produced via synthetic protocols. These particles exist in an unbound state or as aggregates, ranging from 1 nm to 100 nm [106]. The nano-sized nanoparticles allow more current to flow through their cross-section. This feature increases the affectability of the biosensor in comparison with conditions without the application of nanoparticles [28]. Because of the large specific surface area, nanoparticles can detect ultra-low samples. Hence, the affectability and selectivity of molecular diagnosis can be increased by using nanotechnology in clinical assays, as well as its integration with other sciences [28]. Nanomaterials can be used as enzymes, markers, and signal detectors [41]. Among several nanoparticle types, three popular nanoparticles (reduced graphene oxide (RGO), gold nanoparticles (AuNPs), and carbon nanotubes (CNTs)) have been used for electrode surface modification [59]. Nanoparticles have received special attention in the development of electrochemical biosensors for the enrichment of specimens (e.g., magnetic nanoparticle); for their high diagnostic capacity; and as the driver of chemical reactions, the booster of the electrode’s conductance capacity, and the enhancer of biomaterial attachment to the electrode [59].

##### Conjugation of Biomaterials with Au

Gold-based nanostructures for electrical sensing methods were set up in 2001 [107]. One of the most popular nanoparticles that have been widely utilized in biosensors is the AuNP, owing to its unique properties, such as its small-size, catalytic activity, prominent stability, and biocompatibility [59,108]. The surfaces of AuNPs are also rich in negative charges that make them good candidates for tagging a variety of biomolecules [41]. Due to the ease of the immobilization process of nucleic acid on gold electrodes, they have been widely used in the development of biosensors [34]. The conjugation of functionalized groups is performed in different ways, including direct linkage by hydrophobic or electrostatic strength. For example, the direct interaction of proteins and gold, or the indirect linkage achieved by inserting active groups into the structure of molecules such as –SH, is common [35]. The interaction of Au-DNA is controlled by a variety of forces, including non-covalent interactions such as electrostatic or hydrophobic strength, and covalent interactions such as the reaction of functional groups with the bases of nucleotides and gold [35]. These functional groups can be chemical linkers for instance sulfur combinations, such as –SH groups [35]. Davis et al. reported an electrochemical biosensor for fast screening of L.M. using AuNPs and biotinylated capture antibodies. The target analyte can be determined using a urease-labeled detection antibody with a detection limit of 2 log CFU/g [57].

##### Carbon-Based Nanomaterial

Due to their great chemical stability; excellent electrical conduction; biofitness; extraordinary mechanical rigidity; and ability to conform sp, sp2, and sp3 structures with low gaps between the 2s and 2p electron layers of carbon-based nanoparticles, carbon-based nanomaterials are used for the sensitive detection of biocomponents [109].

Graphene oxide

Graphene is a carbon-based material. In 1985, Robert Curl et al. introduced it to the scientific society [110]. It showed considerable properties, such as electrical flexibility, electrical conductivity, and high plasticity [111]. Therefore, researchers developed 2D structures with different and novel properties [111]. D.C. Vanegas et al. developed an aptasensing method using graphene oxide, nanoplatinum, and Pt/Ir electrodes as a recognition layer for better electrochemical results. They used an aptamer for the detection of internalin A, a *Listeria* surface protein. This biosensor exhibited 100 CFU/mL as the limit of detection. The detection procedure lasted 3 h [112].

Carbon nanotube

**Figure 5:**
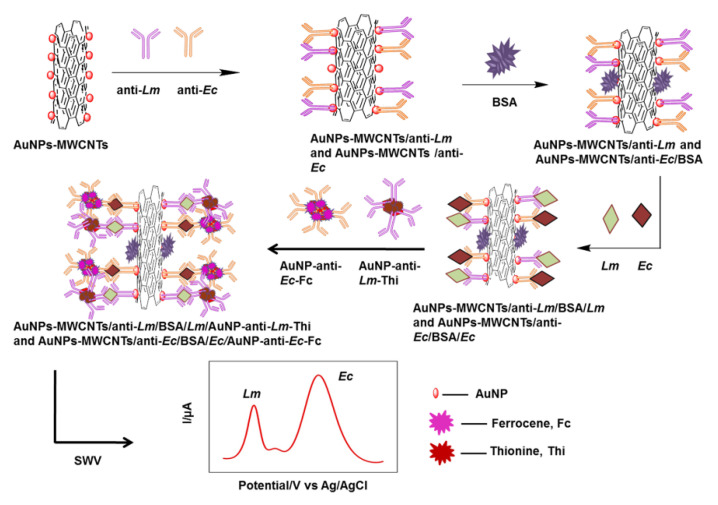
An example of a biosensor that used Au nanomaterials composed of multi-walled carbon nanotubes to increase detection sensitivity [113].

These nanoparticles were first discovered in 1991. Because of their attractive properties, such as exceptional flexibility, high potency for electron transmission, and thermal conduction, carbon nanotubes have earned widespread consideration in the development of biosensors [59]. Lu et al. designed an amperometric biosensor using multi-walled carbon-nanotube fibers (MWCNT) on the functionalized electrode surface. They used an anti-L.M. antibody as a bioreceptor and HRP as a reporter. In this study, different methods of modification were used to optimize the experiment condition. According to the obtained data, the chemical modification method yielded the best sensing outcomes. A detection limit of 1.07 × 10^2^ CFU/mL was achieved in a linear range of 10^2^ to 10^5^ CFU/mL (R2 = 0.993). The developed biosensor was eligible to discriminate L.M. from the rest of the bacterial population in milk samples [46]. In an experiment conducted by Viswanath et al., they applied an electrochemical immunosensor for probing L.M. and *Enterobacter cloacae* in food samples. They used a multi-walled carbon-nanotube-modified Au nanomaterial (AuNPs–MWCNT) nanostructure as the detection platform (Figure 5). The reported linear range of this biosensor was 10^1^–10^11^ for L.M. and 10^1^–10^12^ CFU/mL for *Enterobacter cloacae*. This sensing method showed 3.98 and 5.39 CFU/mL as the limits of detection for L.M. and *Enterobacter cloacae,* respectively [113]. Most types of nanoparticles that were applied for the evaluation of *Listeria* are shown in Table 5.

### 5.2. DNA-Based Signal Amplification Method

In nucleotide-based sensors, DNA is usually used for signal amplification [92]. The conventional real-time PCR assay, as a nucleic acid-based signal amplification technique, is a powerful method, but it has some limitations. For instance, this technique requires an advanced facility, such as a thermal cycler; thus, the loop-mediated isothermal amplification (LAMP) method is an alternative technique. In the LAMP method, the amplification reaction is initiated at an isothermal temperature without the need for a thermocycler. This technique uses the ability of certain DNA polymerases to replace strand activity [115,116]. A summary of the analytical features of a signal amplification method for the detection of *Listeria* is presented in Table 6.

## 6. Conclusions

L.M., as a food-borne bacterium, can grow and survive in environments with varying temperatures and pH values. Despite splendid achievements in the therapeutic regimes associated with listeriosis, L.M. detection remains a significant challenge in the clinical setting. Biosensors play a vital role in the precise and rapid detection of contaminated foods and infected persons. Different types of electrochemical biosensors have been used in the past for analyzing L.M., with significant outcomes. Compared to conventional laboratory analyses, electrochemical biosensors can be used for the in situ detection of L.M. in unprocessed samples with appropriate sensitivity and specificity. In the near future, we will witness the advent and progression of the application of electrochemical biosensors for detecting bacterial particles in clinical settings and industries. In the future, merging electrochemical biosensors with a microfluidic approach will be more applicable to on-desk devices for online monitoring of L.M. It should not be forgotten that some challenges exist in the development of electrochemical biosensors, including their stability and the balance between sensitivity, response time, loss of true activity in real samples, and integration turbulence, which are problematic factors in coordinating electrochemical sensors both at the lab scale and in industry settings. Finding solutions to resolve these problems could allow electrochemical biosensors to be used as in situ detection devices.

## Figures and Tables

**Table 1:** Previously developed electrochemical immunosensors for *Listeria monocytogenes* detection.

Capture	Technique	Plan of Action	Sample	Interfering Agent	LOD	DT *	Ref
Gold nanoparticle—polyclonal antibody	Impedometry	Free-microelectrode	*Listeria* cells	Other bacteria	300 CFU/mL	NR	[42]
HRP-tagged anti-*Listeria monocytogenes* antibody	Amperometry	Functionalized multi-walled carbon nanotube	Milk	Other bacteria	1.07 × 10^2^ CFU/mL	NR	[46]
Antibody against *Listeria* monocytogenes	Amperometry	Antibody-conjugated polypyrrole on platinum electrode	*Listeria* cells (alcohol-killed cells)	*E. coli* and salmonella	10^5^ cells/mL	30 min	[47]
Antibody against *Listeria* monocytogenes	Impedometry	Modified screen-printed gold electrode with SAMs	*Listeria*-containing solution	mouse IgG	Not reported	NR	[48]
Gold nanoparticle –polyclonal antibody	Impedometry	Screen-printed circle-shaped interdigitated gold electrodes (three-finger)	Spiked sample (lettuce)	*E. coli* O157:H7	1.6 × 10^3^ CFU/mL	NR	[49]
Gold NPs-polyclonal anti-*Listeria* monocytogenes antibody conjugate	Impedometry	Microfluidic chip containing interdigitated gold microelectrodes	Lettuce, milk, ground beef	NR	10^4^ and 10^5^ CFU/mL	3 h	[50]
Anti-*Listeria* monocytogenes P60 monoclonal antibody	Amperometry	Modified screen-printed carbon electrodes with anti-P60 protein	Spiked milk	NR	1.5 ng/mL	<3 h	[45]
Anti-*Listeria* monocytogenes p60 antibody	Potentiometry	Eight gold screen-printed electrodes modified by membrane-modified African monkey cells with AntiP60	Cultured *Listeria* bacteria	NR	10^2^ CFU/mL	3 min	[51]
Anti-*Listeria* monoclonal antibody	Chronoamperometry	Functionalized gold electrode with mercaptopropionic acid thiol-SAM	Spiked milk	NR	10^3^ to 10^6^ CFU/mL,	NR	[52]
Biotinylated anti-internalin B polyclonal antibody	Impedometry	Electrical polymerized neutravidin-polyaniline on planar screen-printed carbon electrodes	The recombinant internalin F3 fragment	anti-IgG antibody	4.1 pg/mL	NR	[53]
Anti-*Listeria* monocytogenes monoclonal antibody	Impedometry	Modified gold microelectrode with TiO_2_-nanowires	Cultured bacteria	NR	10^2^ CFU/mL	1 h	[54]
Anti-*Listeria* monocytogenes monoclonal antibody	Impedometry	Modified Au electrode with 11-mercaptoundecanoic acid as a SAM	Filtered tomato extract	NR	4 CFU/mL	NR	[55]
Anti-listeriolysin O antibody	Impedometry	Magnetosomes-Anti-LLO modified screen-printing carbon electrode	Contaminated milk and water	NR	10^1^ Cfu/mL	30-min	[56]
Anti-*Listeria* monocytogenes antibody	Amperometry	Functionalized screen-printed carbon electrode (SPCE) with gold nanoparticles	Blueberry samples	E coli O157:H7 and Salmonella Typhimurium	2 log CFU/g	NR	[57]

* Abbreviations: detection time (DT), self-assembled monolayer (SAM), not reported (NR).

**Table 2:** Genosensors have been used for the detection of *Listeria monocytogenes*.

Capture	Technique	Plan of Action	Sample	Interfering Agent	LOD	Detection Time	Ref
Antibodies (polyclonal IgG) and a DNA aptamer (47-mer)	Impedometry	Chitosan nanobrushes on graphene/nanoplatinum electrodes	Foods	Other Gram-positive bacteria	Immunosensor (15.6 CFU mL^−1^) Aptasensor (9.1 CFU mL^−1^	17 min	[8]
47-mer aptamer against internalin A	Potentiometry	The polycation-responsive membrane	Spiked coastal seawater	High concentration of *Listeria* (2000 CFU mL^−1^)	10 CFU mL^−1^	0.67 h	[11]
24 oligonucleotides of hlyA gene ssDNA	Impedometry	Electropolymerized 5-carboxyindole on gold electrode	Gene extraction product from *Listeria*	NR	2.34 × 10^−13^ M	NR	[39]
24-mer single-strand DNA probe complementary to hlyA gene	Impedometry	Platinum nanoparticles dispersed in the chitosan field	Milk samples	NR	1 × 10^−12^ M to 1 × 10^−4^ M	NR	[69]
59-base inlA gene probe	Potentiometry	Screen-printed gold electrodes decorated with a mercaptan-activated self-assembled monolayer	Culture of *Listeria* monocytogenes	Non-pathogenic *Listeria* species DNA	NR	50–60 min	[72]
30-mer carboxylated probe	Amperometry	Silanization aluminum interdigitated electrode	Target DNA	NR	1 fM to 1 µM	1 h	[16]
Thiolated 49-mer *Listeria* hlyA gene probe	Impedometry	Gold electrodes activated by mercaptan, N-hydroxysulfosuccinimide (NHS), and N-(3-dimethylamion)propyl-N’-ethyl carbodiimide hydrochloride (EDC)	PCR product (Hly gene fragments)	NR	Not reported	NR	[73]
18 thiol-treated oligonucleotides inlA *Listeria* toxin probe	Potentiometry	Screen-printed electrode using carbon and gold-based ink modified with the thiolated probe	*Listeria* inlA amplicon	Non-specific gene fragments	1.5 to 3.6 µM	Less than 1 h	[74]
Amino-treated *Listeria* hly ssDNA probe	Potentiometry	Carbon ionic liquid electrode decorated by reduced graphene and gold nanostructures	PCR product of fish-isolated *Listeria* gene	Mismatch sequences	2.9 × 10^−13^ mol/L	NR	[75]
Amino- 5′- end ssDNA	Potentiometry	Carbon ionic liquid electrode modified by Au nanoparticles and partially-reduced graphene oxide (p-RGO)	Synthetic DNA oligonucleotide	Mismatch sequences	3.17 × 10^–14^ mol/L	NR	[76]
*Listeria* actA gene ssDNA	Potentiometry	Modified gold electrode with mercaptoacetic acid	PCR product	Not reported	NR	NR	[77]
Aminated Hly gene sequences as ssDNA probe	Potentiometry	Self-assembled mercaptoacetic acid (MAA) monolayer on carbon ionic liquid electrode functionalized with three-dimensional graphene rods (3DGR) and gold (Au) nanostructures	PCR product of hly *Listeria* gene amplification	Mismatched sequences	3.3 × 10^−15^ mol/L	NR	[78]
217-mer-biotinated-PCR amplicon of prfA gene	Amperometry	Silica magnetic particles	Streptavidin– horseradish peroxidase	NR	0.13 ng/mL	3 h	[71]
Aminated 20-mer plcA gene sequence	Impedometry	Carbon nanofiber modified gold nanoparticles	Raw milk	Other food-borne bacteria	82 fg/6 µL	30 min	[79]
Aminated ssDNA (ssrA gene)	Amperometry	Decorated gold electrode with cysteamine as a self-assembled monolayer and (osmium-based) redox polymer	Biotinylated ssDNA	NR	1.4 fmol	1 h	[80]
Tetrahedral structure of DNA probe	Amperometry	Bare gold electrode	*Listeria* monocytogenes extraction genome	NR	0.2 fM	~2 h	[81]

**Table 3:** Common working electrodes have been used for the detection of L.M.

Working Electrode	Modified Method	LOD	Detection Time	Ref
Aluminum interdigitated with 1 µm gap-width (Al IDE)	Silanization of the electrode by APTES and immobilization of ssDNA with gold nanomaterials	10 fM to 10 µM	NR	[87]
4 and 8 screen-printed electrodes using carbon and gold-based ink	NR	1.5 to 3.6 µM	Less than 1 h	[74]
Carbon ionic liquid electrode	NR	2.9 × 10^−13^ mol/L	NR	[75]
Gold electrode	Use of conducting polymer (5-carboxyindole)	2.34 × 10^−13^ M	NR	[39]
Gold electrode	methylene blue tagged ssDNA	0.68 aM	45 min	[30]
Screen-printed gold electrodes	Thioctic acid, mercaptopropionic acid, and mercaptoundecanoic acid self-assembled monolayers	Not reported	NR	[48]
Gold electrode	NR	NR	NR	[77]
Microfluidic/microelectrode conjugated system	Magnetic nanoparticle, monoclonal anti-*Listeria*	1.6 × 10^2^ CFU/mL	~1 h	[91]
Screen-printed carbon electrode (SPCE) strips	Anti-*Listeria* conjugated gold nanoparticle	2 log CFU/g	NR	[57]
Screen-printed interdigitated gold electrode	Polyclonal antibody and urease	1.6 × 10 ^3^ CFU/mL	NR	[49]
Screen-printed interdigitated gold electrode	Anti-P60 *Listeria* protein, engineered membrane cells	10^2^ CFU/ mL	3 min	[51]
Planar screen-printed carbon electrodes	Electrical polymerized neutravidin–polyaniline	4.1 pg/ml	NR	[53]
Screen-printed carbon electrode	Conjugation of Magnetosomes and anti-LLO	10^1^ CFU/ml	30 min	[56]
Screen-printed gold electrode	Endolysine, self assembled monolayer	1.1 × 10^4^ and 10^5^ CFU/mL	NR	[58]

**Table 4:** Various labels have been utilized in sensors for the detection of L.M.

Capture	Technique	Sample	Reporter	LOD	Ref
20-mer aminated ssDNA(ssrA gene) of *Listeria*	Amperometry	Solution of target DNA	Glucose oxidase	0.2 nmol	[95]
Amino-treated *Listeria* hly ssDNA probe	Potentiometry	PCR product of deteriorated fish-isolated *Listeria* gene	Methylene blue	2.9 × 10^−13^ mol/L	[75]
Amino- 5′-end ssDNA	Potentiometry	Synthetic DNA oligonucleotides	Methylene blue	3.17 × 10^−14^ mol/L	[76]
Synthetic probe of *Listeria* actA gene	Potentiometry	PCR product of actA gene	Toluidine blue (TB)	NR	[77]
Amino-treated *Listeria* hly ssDNA probe	Potentiometry	PCR product of hly *Listeria* gene amplification	Methylene blue	3.3 × 10^−15^ mol/L	[78]
Gold nanoparticle—polyclonal antibody	Impedometry	*Listeria* cells	Urease	300 CFU/mL	[42]
Amino-treated *Listeria* ssrA gene-probe	Amperometry	Biotinylated DNA strand	Glucose oxidase	1.4 fmol	[80]
Tetrahedral structure of DNA probe	Potentiometry	*Listeria* monocytogenes extraction genome	Ferrocene (Fc)	0.2 fM	[81]
Anti-*Listeria* antibody	Amperometry	Blueberry samples	Horseradish peroxidase	2 log CFU/g	[57]
Anti-*Listeria* antibody	Amperometry	*Listeria* cells	Toluidine blue (TB)	10^5^ cells/mL	[47]
Anti-p60 antibody	Amperometry	Spiked milk	Alkaline phosphatase	1.5 ng mL^−1^	[45]
ssDNA	Amperometry	Extracted genome	Methylene blue	0.68 aM	[30]

**Table 5:** Some of the nanoparticles that were used for the development of L.M.-detecting biosensors.

Capture	Technique	Applied Nanomaterial	LOD	Detection Time	Ref
InlA aptamer	Impedimetry	Graphene/nanoplatinum on Pt/Ir electrodes	100 CFU/mL	3 h	[112]
Amino-treated *Listeria* hly ssDNA probe	Potentiometry	Reduced graphene/nanogold on carbon ionic liquid electrode	2.9 × 10^−13^ mol/L	NR	[75]
Amino- 5′- end ssDNA	Potentiometry	Gold nanoparticle/partially reduced graphene oxide on carbon ionic liquid electrode	3.17 × 10^−14^ mol/L	NR	[76]
HRP-tagged anti-*Listeria* antibody	Amperometry	Multi-walled carbon nanotube	1.07 × 10^2^ cfu/mL	NR	[46]
Anti-*Listeria* antibody(monoclonal)	Impdometry	TiO_2_-Nanowires	10^2^ CFU/mL	1 h	[54]
P100 bacteriophage	Impdometry	Modified carbon nanotubes with quarternized polyethyleneimine	8.4 CFU/mL		[86]
InlA aptamer	Impdometry	PH-sensitive–electroactive ALG-thiomer/Pt nanobrush	(5 CFU mL^−1^)	17 min	[64]
47-mer aminated aptamer	Impdometry	WS2 nanoparticles	10 CFU/mL	NR	[114]

**Table 6:** Summarized data of DNA- based signal amplification method for *Listeria* detection.

**Capture**	**Technique**	**Signal Amplification Method**	**LOD**	**Ref**
Amino-treated ssDNA	Amperometry	Double linear hybridization chain reaction (DL-HCR)	1.64 × 10^4^ CFU/mL and 11 CFU/mL	[70]

## Data Availability

All required data are presented in this study.
